# Perceived Neighborhood Racial Composition and Depressive Symptoms Among Black Americans Across Adulthood: Evaluating the Role of Psychosocial Risks and Resources

**DOI:** 10.1177/08982643221100789

**Published:** 2022-06-03

**Authors:** Courtney S. Thomas Tobin, James Huynh, Heather R. Farmer, Rebekah Israel Cross, Apurva Barve, Millicent Robinson, Erika Perez Leslie, Roland J. Thorpe

**Affiliations:** 1 Department of Community Health Sciences, 25808 University of California, Los Angeles, CA, USA; 2 Department of Human Development and Family Sciences, 5972 University of Delaware, Newark, DE, USA; 3 Department of Health Behavior/ Department of Maternal and Child Health, 25808 University of North Carolina Gillings School of Global Public Health, Chapel Hill, NC; 4 School of Social Work, 25808 University of North Carolina at Chapel Hill, Chapel Hill, NC; 5 Metro Nashville Public Health Department, 461266 Nashville, TN; 6 Program for Research on Men’s Health, 25802 Hopkins Center for Health Disparities Solutions, Baltimore, MD, USA

**Keywords:** Perceived neighborhood racial composition, residential segregation, depressive symptoms, stress exposure, psychosocial resources, ethnic density, black Americans

## Abstract

**Objectives:** To evaluate the relationships between perceived neighborhood racial composition (PNRC), psychosocial risks and resources, and depressive symptoms among young (ages 22–35), middle-aged (ages 36–49), and older (ages 50+) Black Americans. **Methods:** Full sample and age-stratified linear regression models estimated the PNRC-depressive symptoms association and the extent to which it persisted after accounting for psychosocial risks (i.e., neighborhood disorder, other social stressors) and resources (i.e., mastery, social support, racial identity) among 627 Black Americans in the Nashville Stress and Health Study. **Results:** Living in racially integrated and predominately White neighborhoods was associated with elevated depressive symptoms. While psychosocial risks and resources explained a substantial portion of these associations, patterns varied across age groups. **Discussion:** PNRC impacts depressive symptoms among Black Americans by shaping psychosocial risks and resources. Findings underscore interconnections between contextual and psychosocial factors, as well as the distinct mental health significance of these processes across stages of adulthood.

## Introduction

Depression is a major public health concern in the United States. This is especially true among aging populations of color, as life course theories suggest that minoritized racial status may further exacerbate risk for some groups, including older Black Americans (Brown et al., 2016; Murray et al., 1989; Thomas Tobin et al., 2020). Although epidemiological studies demonstrate that Black Americans experience relatively low rates of major depressive disorder across the life course (Barnes & Bates, 2017; Louie et al., 2021; Thomas Tobin et al., 2020), there is also ample evidence that Black individuals tend to endorse severe and disabling depressive symptoms (Wyman et al., 2020; Wang et al., 2000; Williams & Earl, 2007; Walton & Shepard Payne, 2016) that are linked to co-occurring chronic physical health conditions (e.g., hypertension, diabetes, and heart disease) and contribute to the elevated rates of premature mortality of this group (Newcomer, 2007; Pickering, 2000; Williams & Earl, 2007). At the same time, the distribution of depressive symptoms across the life course is well-documented, with young and older adults typically experiencing more depressive symptoms than their middle-aged counterparts (Hargrove et al., 2020; Mirowsky & Ross, 2017; Sutin et al., 2013). Prior research underscores the significance of life stages for shaping individuals’ experiences and exposures, which are directly linked to age and cohort differences in mental health (Hargrove et al., 2020; Thomas Tobin et al., 2022). Thus, the risks associated with depressive symptoms among Black Americans may vary significantly across stages of adulthood. Given that depressive symptoms have been linked to significant social and personal burdens (e.g., poor physical health, strained interpersonal relationships, limited educational and labor market productivity) (Hargrove et al., 2020), and recent findings point to distinct causal pathways to depressive symptoms among Black Americans (Thomas Tobin, 2021), identifying the specific factors contributing to heightened symptomatology across young, middle, and older adulthood is needed to improve outcomes among this population.

Despite a recent increase in research on psychiatric disorders among older adults, fewer have investigated the risk and protective factors that shape the depressive symptoms of Black Americans across the life course (Hargrove et al., 2020; Mouzon et al., 2017). In addition, most studies on social inequalities in mental health tend to emphasize individual-level characteristics such as socioeconomic status (SES). However, since Black Americans report similar levels of depressive symptoms across SES levels (Assari, 2018; Williams & Neighbors, 2001; Williams et al., 1992), there has been growing interest in the role of contextual and psychosocial factors, which may account for elevated rates of depressive symptoms independent of socioeconomic resources. For instance, scholars have argued that place, and neighborhood conditions, more specifically, may better explain persistent health inequalities than race alone (LaVeist et al., 2011; Mezuk et al., 2013). Yet, the specific ways that neighborhood contexts shape mental health across the life course among Black Americans have been underexplored in prior research.

The present study addresses these issues by investigating the mental health significance of one indicator of neighborhood context, perceived neighborhood racial composition (PNRC). PNRC refers to individuals’ subjective assessment of the racial composition of their communities (Hidalgo et al., 2015). PNRC may provide insight into the ways that the concentration of racially minoritized individuals within neighborhoods create distinct sociocultural environments that influence health (Acevedo-Garcia & Lochner, 2003; Kwate, 2008; Massey, 2004). We draw on the stress process model (Pearlin et al., 1981), a popular framework used to assess social inequalities in mental health, to better understand how PNRC shapes depressive symptoms among Black Americans across the life course. A central tenet of this framework is that inequalities in mental health arise from differential exposure to social stressors and access to protective psychosocial resources, which are patterned by the contexts and conditions in which individuals live (Pearlin et al., 1981). As such conditions of life are influenced and often constrained by one’s social identities (i.e., race, SES, gender), neighborhood contexts represent a critical mechanism through which the mental health of Black Americans is shaped and may contribute to adverse outcomes among this population. This is further underscored by life course perspectives, which emphasize the ways that individuals accumulate health risks and resources as they age and experience various stages of adulthood, as well as the influence of time and place in shaping these processes (Pearlin et al., 2005). Therefore, we integrate these perspectives to assess the links between PNRC, psychosocial risks and resources, and depressive symptoms among Black Americans and evaluate the extent to which these relationships vary across young, middle, and older adulthood.

## Background

### Neighborhood Contexts and Mental Health

The importance of neighborhood contexts for shaping mental health is well-documented. Studies have examined neighborhood contexts with objective and subjective indicators that assess the physical, cultural, economic, and political environment, as well as how individuals perceive or experience the environment (Hill & Maimon, 2013). Prior research has shown a strong association between neighborhood contexts and depressive symptoms across the life course, highlighting the significance of structural and economic factors such as low neighborhood SES, graffiti, noise, pollution, aggressive policing, and crime (Aneshensel et al., 1991; Gee & Payne-Sturges, 2004; Ross & Mirowsky, 1999; Sewell, 2016). Though such conditions tend to be more prevalent in socioeconomically disadvantaged communities, due to the historical and contemporary hypersegregation of Black Americans in resource-poor communities, Black people at all levels of SES are more likely than their White counterparts to live in more socially disadvantaged and predominately Black neighborhoods (Brenner et al., 2013; Massey & Denton, 1989, 1993; Merkin et al., 2009; Williams & Williams-Morris, 2000). However, some studies show that White and Black individuals living in the same communities often experience comparable health outcomes, an indication that the economic and racial patterning of residential segregation shapes exposure to risks that produce racial health disparities across the life course (LaVeist et al., 2011; Thorpe et al., 2016). Prior work also suggests that for many Black Americans, poor community conditions may lessen the mental health benefits associated with greater individual-level SES (Hudson et al., 2013; Lacy, 2007; Thomas, 2015). Consequently, additional research is needed to identify the characteristics of neighborhood contexts beyond structural and economic conditions that are most influential for the mental health of Black Americans.

### PNRC and Mental Health

Perceived neighborhood racial composition (PNRC), or the subjective evaluation of the racial makeup of a community, is one dimension of neighborhood context with significant implications for mental health outcomes among Black Americans (Burton & Jarrett, 2000; English et al., 2014; Leventhal & Brooks-Gunn, 2003). Since it provides information about the general proportion of individuals from various racial groups within a community, PNRC is often used as an indicator of residential segregation (Anglin et al., 2020; Ray, 2017). Unlike other segregation measures, however, PNRC may provide additional insights into individuals’ subjective experiences within neighborhoods. For instance, studies suggest that PNRC reflects levels of interracial interactions within a community (Eschholz et al., 2003; Pickett et al., 2012). Prior research also finds that PNRC is related to Black Americans’ preferences when selecting neighborhoods in which to live (Charles, 2000), their social and political attitudes (Stevenson et al., 2005), as well as their feelings of safety within communities (Ray, 2017).

Most importantly, PNRC has been linked to depressive symptoms among Black Americans, although studies have yielded mixed results. Previous findings suggest that the residential racial composition may be of particular importance for the social experiences and health of Black Americans, above and beyond their SES (Lacy, 2007; Pattillo, 1999). For example, some research has shown that living in a neighborhood with a high percentage of Black people, while accounting for SES, is associated with greater depressive symptoms for Black Americans (Bécares et al., 2014; Do et al., 2019; Mair et al., 2010). Yet, others have found that Black Americans who live in predominately White communities tend to endorse more depressive symptoms (English et al., 2014; Vogt Yuan, 2007; Wickrama et al., 2005; Wight et al., 2005. Though mixed, these findings collectively indicate that PNRC matters for depressive symptoms among Black Americans. It is possible that these inconsistent patterns are the result of many studies examining the link between PNRC and mental health among samples aggregated across race and age, which may obscure key subgroup differences (English et al., 2014; Mair et al., 2010; Wickrama et al., 2005). As such, the ways in which PNRC may influence depressive symptomatology among Black Americans at various stages of the life course remains unclear.

Several explanations have been proposed. A large body of evidence suggests that depressive symptom levels would be highest among Black Americans who live in predominately Black neighborhoods, as such contexts facilitate exposure to psychosocial risks that are detrimental for health (Bécares et al., 2014; Do et al., 2019; Riley, 2018). Often referred to as the *racial residential segregation hypothesis*, this perspective argues that residential segregation is a fundamental determinant of health that also promotes psychosocial risks, such as perceived neighborhood disorder and exposure to other social stressors associated with elevated depressive symptoms (Brenner et al., 2013; Diez Roux & Mair, 2010; Kramer & Hogue, 2009; Kruger et al., 2007; Latkin & Curry, 2003; White et al., 2012). Perceived neighborhood disorder is a form of stress that arises when individuals perceive little order and social control within their communities due to issues such as noise, graffiti, crime, crowding, and trouble with neighbors (Mirowsky & Ross, 1999). Given limited socioeconomic resources, the physical condition of many predominately Black neighborhoods may induce perceptions of social disorder among residents (Mirowsky & Ross, 1999; Sewell et al., 2016). Individuals in disadvantaged communities also face greater exposure to general stressors, including acute and chronic stress events, discrimination, and trauma across the life course (Blair et al., 2014; Giurgescu et al., 2015; Pickover et al., 2018). Since past research indicates that Black Americans experience greater social stress relative to their White counterparts (Sternthal et al., 2011; Turner & Avison, 2003), these exposures may be further amplified among those living in predominately Black neighborhoods (Brenner et al., 2013; George & Lynch, 2003; Jackson, & Knight, 2006; Schulz et al., 2008).

While the racial residential segregation hypothesis would also suggest that such contexts may also diminish the availability of protective psychosocial resources that help individuals to combat the negative impact of stressors on health (Giurgescu et al., 2015; Lin et al., 2009), an alternative perspective highlights the benefits of living within predominately Black communities. Known as the *ethnic density hypothesis,* this explanation argues that Black Americans who live in predominately Black neighborhoods may in fact endorse fewer depressive symptoms, because living in communities with other Black people may promote resilience by facilitating the development of protective psychosocial resources (Bécares et al., 2014; Diez Roux & Mair, 2010). Psychosocial resources include both individual-level characteristics (e.g., sense of control) and those that arise from one’s social relationships (e.g., instrumental and emotional support from family members and friends) (Nguyen et al., 2019; Thomas Tobin et al., 2021). In addition, individuals’ racial identity, or sense of collective identity-based on perceptions of sharing a common racial heritage with a particular group (Demo & Hughes, 1990), is also importantly shaped by neighborhood racial composition (English et al., 2014).

A substantial body of research has demonstrated the protective influence of psychosocial resources on mental health among Black Americans (Erving & Thomas, 2018; Hughes et al., 2015; Mouzon, 2013). Prior work also indicates that the availability of these resources varies across social locations and throughout the life course, such that individuals from disadvantaged backgrounds typically report fewer resources (Turner & Roszell, 1994; Williams et al., 2016). Yet, recent studies show that Black Americans report relatively high levels of psychosocial resources relative to other groups (Thomas Tobin & Moody, 2021). Hence, the depressive symptoms of those residing in predominately Black neighborhoods may be diminished due to greater access to protective psychosocial resources such as mastery, social support, and racial identity, which are needed to cope with the stressors and the strains associated with socially disadvantaged communities.

### The Present Study

**Figure 1.**
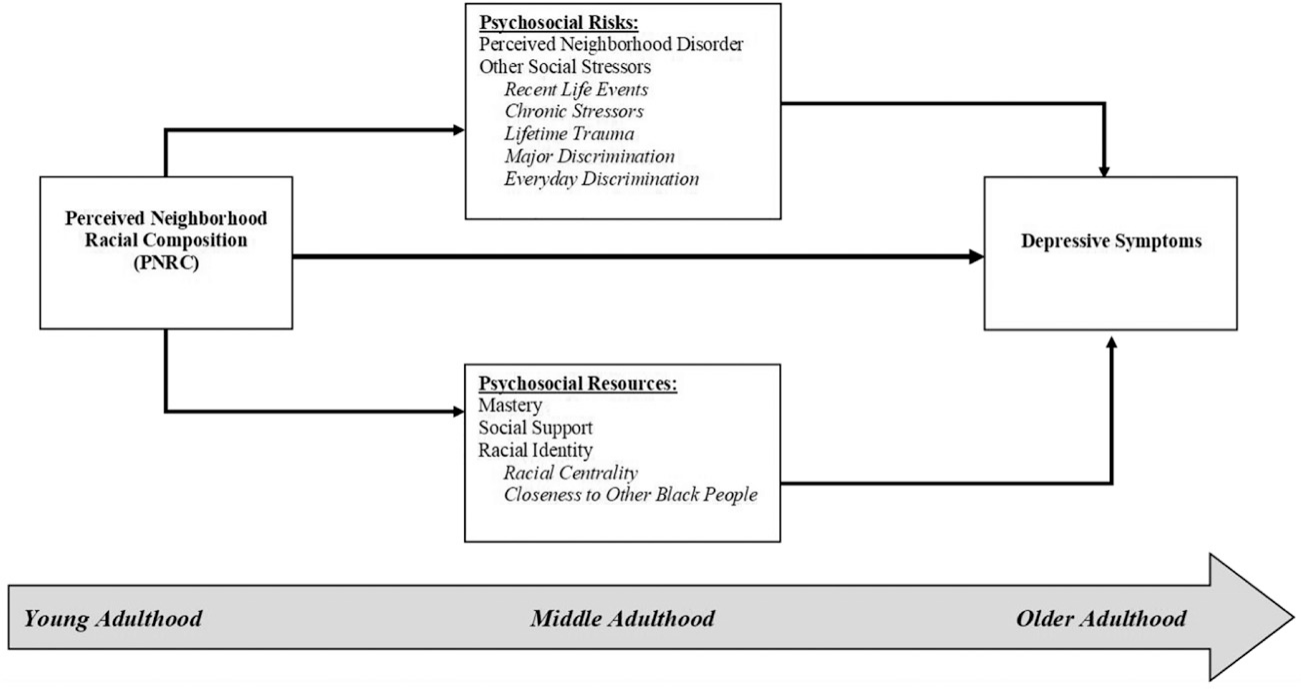
Conceptual model of evaluating the roles of psychosocial risks and resources in the association between perceived neighborhood racial composition and depressive symptoms among young, middle-aged and older black adults.

Considering the inconsistent findings of prior research, the present study aims to clarify the links between PNRC and depressive symptoms among Black Americans across the life course. Integrating the stress process model with life course perspectives in Figure 1, we recognize PNRC as an important indicator of neighborhood context that not only reflects a community’s level of racial residential segregation—and by extension, is indicative of its historical and contemporaneous access to resources and isolation from “mainstream” society (Massey, 2004; Massey & Denton, 1993)—but also the extent to which a neighborhood has been racialized as “White space” (in contrast to “Black space”) and is socially perceived as valued and safe (Anderson, 2015; Lipsitz, 2007). To this end, PNRC may provide important insight into the complex ways in which race (as a social identity) and racism (as a form of systemic oppression) produce inequalities in mental health. Moreover, this lens allows us to conceptualize the specific ways that PNRC shapes depressive symptoms among Black Americans via its influence on psychosocial risks and resources.

Although a large body of research has examined the relationships between various indicators of neighborhood context and mental health (see Hill & Maimon, 2013 for a review), several limitations impede our understanding of the PNRC-depressive symptoms association among Black Americans. One challenge is that previous studies point to the opposing racial residential segregation and ethnic density hypotheses as explanations for this relationship. While both explanations imply that PNRC matters for this group’s mental health because it contributes to greater exposure to psychosocial risks (i.e., racial residential segregation) or resources (i.e., ethnic density), few studies have empirically assessed the direct linkages between these factors and PNRC. As such, their role in the PNRC-depressive symptoms relationship remains unclear.

Another challenge is that there has been relatively little consideration of the ways that these processes vary across the life course, as many studies have aggregated this population across different adult life stages or have exclusively focused on young adults (Aneshensel & Sucoff, 1996; Robert & Ruel, 2006); even fewer have considered older adults (Everson-Rose et al., 2011; Yen et al., 2009). Such approaches likely obscure critical differences in the significance of PNRC for shaping psychosocial risks and resources, since prior research suggests they develop based on the varied experiences of individuals’ lives over time (Turner, 2013). In addition, the cumulative impact of PNRC on psychosocial risks and resources may vary across age groups (Alwin & Wray, 2005) because of the distinct historical racial contexts that they have experienced (Mouzon et al., 2017). For instance, older Black Americans (50+) who grew up during the Civil Rights Movement in the 1960s would have experienced very different neighborhood contexts compared to middle-aged adults (36–49) who grew up during a post-racial ethos, or young adults (22–35) who might have relatively fewer experiences with overt racism. Furthermore, since individuals’ perceptions of neighborhood contexts, exposure to social stressors, and the availability of psychosocial resources all vary significantly with age (Gilbert et al., 2015; Pearlin et al., 2005), it is important to know which of these factors are most influential for mental health at various stages of adulthood.

To address these limitations, the present study will (1) assess differences in psychosocial risks and resources across varied neighborhood compositions (i.e., neighborhoods perceived as mostly Black, half Black, or mostly White); (2) examine the relationship between PNRC and depressive symptoms; and (3) evaluate the extent to which psychosocial risks and resources account for the PNRC-depressive symptoms association among young, middle-aged, and older Black Americans. Our study advances prior work by examining multiple dimensions of psychosocial risks and resources, including neighborhood disorder and other social stressors (i.e., recent life events, chronic stressors, lifetime trauma, lifetime major discrimination, everyday discrimination), in addition to perceived mastery, social support, and racial identity (i.e., racial centrality, closeness to other Black people), all of which have been associated with mental health among Black Americans in prior research (Hughes et al., 2015; Kiecolt et al., 2009; Mouzon, 2013). By clarifying these linkages, we seek to enhance understanding of the ways that PNRC shapes depressive symptoms among Black Americans across the life course and inform more effective strategies to reduce the burden of depression in this population.

## Method

### Sample

The Nashville Stress and Health Study (NSAHS) is a population-based sample of community-dwelling, non-Hispanic Black and White adults of ages 21 to 69 years from the city of Nashville and surrounding areas within Davidson County, Tennessee. A random sample was obtained using a multistage, stratified sampling approach. Black households were oversampled to achieve a final sample with similar proportions of racial and sex groups, and a sampling weight allowed for generalizability of sample characteristics to the county population. Between 2011 and 2014, 1252 respondents, including 627 Black Americans, were successfully interviewed about their personal and family backgrounds, stress and coping experiences, health behaviors, and health histories during three-hour computer-assisted interviews with trained study staff of the same race. All participants provided informed consent. Study procedures were approved by the Vanderbilt University Institutional Review Board and have been described in detail elsewhere (see Brown et al., 2016). The present analyses include data from 627 Black respondents, and sample characteristics are shown in Table 1.

**Table 1.** Sample Characteristics by Age among Black Americans, Nashville Stress and Health Study (2011–2014).

	All (*N* = 627)		Young adults (*n* = 146)		Middle-aged adults (*n* = 208)		Older adults (*n* = 273)	
	22–69 years		22–35 years		36–49 years		50+ years	
Depressive symptoms (CES-D) [0–47]	14.33	(12.58)	16.55b^c^	(10.16)	14.47^a,c^	(13.67)	12.24^a^	(12.89)
Perceived neighborhood racial composition								
Mostly Black (Ref.)	44.42		54.35^b,c^		41.57^a^		38.16^a^	
Half Black	28.71		26.29		31.82		28.07	
Mostly White	26.88		19.36b^c^		26.61^a^		33.77^a^	
Psychosocial risks								
Perceived neighborhood disorder [12–47]	23.29	(8.75)	24.88^c^	(8.24)	23.00	(9.17)	22.69a	(8.02)
Other social stressors [−1.31–3.56]	0.14	(0.92)	0.27^c^	(0.74)	0.13	(0.83)	0.03a	(1.11)
Psychosocial resources								
Mastery [13–35]	26.62	(6.80)	27.51^c^	(5.35)	26.80	(7.54)	25.65^a^	(7.11)
Social support [−2.84–0.86]	−0.02	(1.02)	0.02	(0.75)	−0.08	(1.15)	−0.005	(1.14)
Racial centrality [5–28]	26.08	(4.17)	25.46	(4.19)	26.42	(3.69)	26.33	(4.16)
Closeness to other Black people [6–42]	33.72	(8.80)	32.20^b,c^	(7.46)	34.00^a^	(8.02)	34.83^a^	(10.22)
Sociodemographic characteristics								
Gender								
Women (Ref.)	54.90		57.83		51.62		55.24	
Men	45.10		42.16		48.38		44.76	
Socioeconomic status (SES) [−2.93–1.68]	−0.47	(0.97)	−0.30	(0.78)	−0.53	(1.02)	−0.58	(1.05)

*Notes.* Weighted means or percentages are presented; variable ranges included in brackets; "Young Adults" is the reference category (Ref.).

a significantly different from Young Adults, *p* < .05.

b significantly different from Middle-Aged Adults, *p* < .05.

c significantly different from Older Adults, *p* < .05.

### Measures

#### Depressive Symptoms

Past-month depressive symptomatology was assessed using the 20-item (Cronbach’s alpha = 0.89) Center for Epidemiologic Studies Depression Scale (Radloff, 1977). Examples of items included “could not shake off the blues,” “felt depressed,” “sleep was restless,” and “had crying spells.” Response categories ranged from (0) not at all to (3) very much. Items were summed, such that scores ranged from 0 to 60, with higher values indicating higher depressive symptoms.

#### Perceived Neighborhood Racial Composition

A single-item measure was used to assess respondents’ subjective evaluations of the relative proportions of Black and White people in their neighborhoods (Chiricos et al., 1997; 2001). The following prompt was provided: “Think about the places you have lived, gone to school, or work—were mostly Blacks or mostly Whites there?” Respondents were then asked to rate their present neighborhood on a 5-point Likert scale: (0) All Blacks, (1) Mostly Blacks, (2) About Half Blacks, (3) Mostly Whites, (4) Almost All Whites. However, to avoid the assumption that PNRC operates in a linear fashion and since prior research suggests that neighborhoods perceived as “all Black” or “mostly Black” would be similarly racialized as a “Black space,” just as those perceived as “all White” or “mostly White” would be similarly racialized as “White space” (Anderson, 2015; Lipsitz, 2007), responses were collapsed into the following categories (0) Mostly Black [reference category], (1) About Half Black, (2) Mostly White.

#### Psychosocial Risks

Two forms of psychosocial risk were assessed. *Perceived neighborhood disorder* (Ross & Mirowsky, 1999) was assessed using 13 items (α = 0.90) about the cleanliness, safety, and social interactions within respondents’ neighborhoods. Items included “There is a lot of graffiti in my neighborhood,” “There are too many people hanging around on the streets near my home,” and “I’m always having trouble with my neighbors.” Responses ranged from (1) strongly disagree to (4) strongly agree and were summed such that higher scores corresponded with high levels of disorder. Since prior research argues for the comprehensive assessment of social stress exposure as not to underestimate its significance for mental health among Black Americans (Turner & Taylor, 2002; Sternthal et al., 2011), the present study also evaluated five dimensions of *other social stressors*: recent life events, chronic stress, trauma, everyday discrimination, and major discrimination (see Turner, 2013 for description of individual stress scales). Consistent with previous studies (Brown & Hargrove, 2018; Thomas Tobin, 2021; Turner, 2013; Turner & Taylor, 2002), we created a composite index of social stress exposure to capture individuals’ exposure to a variety of stressors, including both acute and chronic experiences, across the life course. To measure total stress exposure, each dimension was standardized and summed so that 0 represented the mean; scores less than 0 indicated below average exposure and scores above 0 indicated higher than average exposure.

#### Psychosocial Resources

We also examined four psychosocial resources in this study. *Mastery* was measured using Pearlin and Schooler’s (1978) 7-item (α = 0.70) Perceived Mastery Scale (e.g., “You have little control over the things that happen to you”). Responses ranged from (1) *strongly agree* to (5) *strongly disagree* and were summed so that higher scores indicated higher mastery. Perceived *social support* was based on two measures of support from family and friends. Family support measured individuals’ perceptions of being able to rely on family for emotional and instrumental support in times of need. Eight items such as “You feel very close to your family” and “No matter what happens you know that your family will always be there for you should you need them” were used. The same items were adapted to examine perceived friend support (Turner & Marino, 1994). Responses ranged from (1) *not at all true for* you to (4) *very true for you*. Reliability was high for both family (α = 0.92) and friend (α = 0.95) support measures. Items were standardized and summed so higher scores indicated greater support from family and friends.

The NSAHS also assessed the extent to which Black Americans identified with their racial heritage (Turner, 2013). Results from confirmatory factor analyses indicated two distinct constructs most closely aligned with the concepts of “racial centrality” and “closeness to other Black [people]” found in the literature (Demo & Hughes, 1990; Sellers et al., 1998). *Racial centrality* evaluated the degree to which individuals feel being Black is central to their self-concept. It was measured with 4 items (α = 0.72) including “You have a strong sense of yourself as a member of your racial/ethnic group.” *Closeness to Other Black People (COBP)* was measured by 6 items (α = 0.78) that assessed the degree to which respondents feel connected to other Black Americans (e.g., “Your values, attitudes, and behaviors are shared by most members of your racial/ethnic group”). Responses for both identity dimensions ranged from (1) *strongly disagree* to (7) *strongly agree.* Items were summed so higher values corresponded with a greater sense of a central Black identity and feelings of closeness to other Black people.

#### Age

*Age* was measured categorically: (0) young adults (22–35 years), (1) middle-aged adults (36–49 years), and (2) older adults (50+ years).

#### Covariates

*Gender* was based on self-report: (0) Women, (1) Men. *Socioeconomic status (SES)* was assessed using a standardized index of years of completed education, annual household income, and level of occupational prestige determined by the 2000 Nam-Powers-Boyd occupational scores (Nam & Boyd, 2004). SES scores were calculated by first standardizing and summing the three dimensions, then dividing by the number of dimensions on which data were available (Erving & Thomas, 2018; Thomas Tobin & Moody, 2021).

### Analytic Strategy

First, weighted means and frequency distributions were calculated for the full sample and separately among age groups; t-tests and *X*^2^ tests determined significant differences across young, middle-aged, and older adults (Table 1). Second, we assessed mean levels of psychosocial risks and resources by PNRC and age in Table 2. Third, we examined the relationship between PNRC and depressive symptoms among the full sample in Table 3. The impact of PNRC on depressive symptoms was assessed in Model 1. Psychosocial risks (perceived neighborhood disorder, other social stressors) were added in Model 2, while psychosocial resources (mastery, social support, racial centrality, COBP) were considered in Model 3. Model 4 assessed the PNRC-depressive symptoms association while accounting for risks and resources simultaneously. Age, gender, and SES were included as covariates in all models.

**Table 2.** Mean Levels of Psychosocial Risks and Resources by Perceived Neighborhood Racial Composition and Age, Nashville Stress, and Health Study (2011–2014).

	All (*N* = 627) 22–69 years			Young adults (*n* = 146) 22 to 35 years			Middle-aged adults (*n* = 208) 36–49 years			Older adults (*n* = 273) 50+ years		
	Mostly Black	Half Black	Mostly White	Mostly Black	Half Black	Mostly White	Mostly Black	Half Black	Mostly White	Mostly Black	Half black	Mostly White
Psychosocial risks												
Perceived neighborhood disorder	25.41^b,c^	23.34^a,c^	20.47^a,b^	26.50^c^	24.57	20.77^a^	25.30^b,c^	22.60^a^	19.87^a^	24.13^c^	23.08^c^	20.73^a,b^
Other social stressors	0.03^c^	0.19	0.26^a^	0.12^b^	0.66^a^	0.15	0.04	0.19	0.20	0.09^c^	0.19^c^	0.35^b,c^
Psychosocial resources												
Mastry	27.11^b^	25.94^a^	26.51	27.56	27.56	27.30	27.56^b^	25.19^a^	27.54	26.11	25.35	25.39
Social support	0.01	−0.03	−0.06	0.04	−0.04	0.06	−0.06	−0.04	−0.14	0.05	−0.001	−0.06
Racial centrality	26.66^b,c^	25.61^a^	25.61^a^	26.34^c^	24.32	24.56^a^	26.60	26.64	25.86	27.14^b,c^	25.63^a^	25.98^a^
Closeness to other Black people	34.64^c^	34.08^c^	31.83^a,b^	32.73^c^	33.16^c^	29.40^a,b^	34.15	35.73^c^	31.69^b^	37.51^b,c^	33.17^a^	33.17^a^

*Note.* Weighted means (with standard deviations in parentheses) of psychosocial risks and resources across categories of perceived neighborhood racial composition are shown.

a significantly different from “Mostly Black,” *p* < .05.

b significantly different from “Half Black,” *p* < .05.

c significantly different from “Mostly White,” *p* < .05.

**Table 3.** Depressive Symptoms Regressed on Perceived Neighborhood Racial Composition and Psychosocial Risks and Resources among Black Adults, Nashville Stress and Health Study (2011–2014; *N*=627).

	Model 1	Model 2	Model 3	Model 4
Perceived neighborhood racial composition				
Mostly Black (Ref.)				
Half Black	3.72**	2.66**	2.53**	1.56*
	(1.25)	(0.97)	(0.87)	(0.75)
Mostly White	3.09**	1.85*	2.19*	0.74
	(1.14)	(0.83)	(0.99)	(0.64)
Psychosocial risks				
Perceived neighborhood disorder		0.11*		−0.05
		(0.05)		(0.06)
Other social stressors		6.80***		5.46***
		(0.43)		(0.43)
Psychosocial resources				
Mastery			−0.71***	−0.56***
			(0.09)	(0.08)
Social support			−2.39***	−1.58*
			(0.69)	(0.65)
Racial centrality			−0.42*	−0.44*
			(0.21)	(0.19)
Closeness to other Black people			0.13	0.11
			(0.08)	(0.07)
Intercept	15.84***	11.15***	42.96***	39.21***
	(1.39)	(2.15)	(4.45)	(4.97)
R^2^	0.14	0.38	0.37	0.51
F	12.00	55.08	79.85	83.81

*Note.* Unstandardized OLS coefficients and standard errors (in parentheses) are presented. Age, gender, and socioeconomic status are included as covariates in all models.

*p < .05, **p < .01, ***p < .001 (two-tailed tests).

To understand the role of the psychosocial factors, we evaluated changes in the PNRC coefficients after controlling for risks and resources. For example, significant reductions in the magnitude of the PNRC-depressive symptoms association (or a reduction to non-significance) when accounting for psychosocial risks in Model 2 would suggest that risk factors at least partially explain the relationship, a pattern in line with the racial residential segregation hypothesis; if accounting for psychosocial resources reduced the PNRC-depressive symptoms association to non-significance, it would suggest resources primarily explain the relationship, which provides support for the ethnic density hypothesis.

A similar approach was used within age-stratified models in Table 4 to evaluate these relationships among young (Models 1–2), middle-aged (Models 3–4), and older adults (Models 5–6). For each age group, the PNRC-depressive symptoms association was examined in the first model; psychosocial risks and resources were added in the second model. *p*-values less than 0.05 were considered statistically significant. All analyses were performed using STATA 17.1.

**Table 4.** Depressive Symptoms Regressed on Perceived Neighborhood Racial Composition and Psychosocial Risks and Resources among Young, Middle-Aged, and Older Black Adults, Nashville Stress and Health Study (2011–2014).

	Young adults (Age 22–35)		Middle-aged adults (Age 36–49)		Older adults (Age 50+)	
	Model 1	Model 2	Model 3	Model 4	Model 5	Model 6
Perceived neighborhood racial composition						
Mostly Black (Ref.)						
Half Black	7.76***	3.10	1.37	−1.60	1.95	2.66**
	(1.71)	(1.65)	(2.12)	(1.37)	(1.40)	(1.00)
Mostly White	3.27*	1.02	1.01	−1.76	4.80***	3.20**
	(1.69)	(1.34)	(1.65)	(1.90)	(1.42)	(1.05)
Psychosocial risks						
Perceived neighborhood disorder		−0.16		−0.10		0.22*
		(0.17)		(0.10)		(0.09)
Other social stressors		4.67***		6.60***		4.74***
		(0.79)		(1.03)		(0.67)
Psychosocial resources						
Mastery		−0.47**		−0.83***		−0.50**
		(0.14)		(0.14)		(0.16)
Social support		−2.98**		0.08		−2.09*
		(0.95)		(0.93)		(0.81)
Racial centrality		−0.70**		−0.35		0.27
		(0.24)		(0.32)		(0.21)
Closeness to other Black people		0.23*		−0.04		0.01
		(0.11)		(0.10)		(0.11)
Intercept	16.71***	42.62***	12.31***	49.50***	10.15***	10.95
	(0.93)	(9.17)	(1.66)	(7.93)	(1.57)	(6.13)
N	146	146	208	208	273	273
R^2^	0.29	0.57	0.12	0.55	0.08	0.49
F	10.00	57.84	5.27	30.38	5.99	57.06

*Note.* Unstandardized OLS coefficients and standard errors (in parentheses) are presented. Age, gender, and socioeconomic status are included as covariates in all models.

*p < .05, **p < .01, ***p < .001 (two-tailed tests).

## Results

### Sample Characteristics

Descriptive statistics of the sample are provided in Table 1. Overall, the average depressive symptoms score was 14.22 (SD = 12.58), which is slightly below the clinical significance threshold of 16 (Lewinsohn et al., 1997). While most respondents (44.42%) reported living in predominately Black neighborhoods, 28.71% described their neighborhoods as “about half Black” and 26.88% as “mostly White.” Exposure to psychosocial risks was generally moderate among Black Americans, such that mean scores for perceived neighborhood disorder and other social stressors were 23.29 (SD = 8.75, range = 12–47) and 0.14 (SD = 0.92, range= −1.31–3.56), respectively. Nevertheless, average levels of mastery (m = 26.62, SD = 6.80, range = 13–35), social support (m = −0.02, SD = 1.02, range = −2.84–0.86), racial centrality (m = 26.08, SD = 4.17, range = 5–28), and COBP (m = 33.72, SD = 8.80, range = 6–42) were relatively high.

Significant age differences also emerged. Depressive symptoms varied across age groups, such that the depressive symptom scores of middle-aged (m = 14.47, SD = 13.67) and older adults (m = 12.24, SD = 12.89) were significantly lower than the scores of young adults (m = 16.55, SD = 10.16). PNRC also varied significantly by age, as more young adults lived in mostly Black communities (54.35%), while more older adults resided in mostly White communities (33.77%). Young adults reported significantly higher exposure to psychosocial risks relative to middle-aged and older adults. Psychosocial resources were more varied. While mastery decreased significantly with age, COBP increased with age, and social support and racial centrality scores were consistent across young, middle-aged, and older adults.

#### Psychosocial Risks and Resources by PNRC and Age

The bivariate associations between PNRC and the psychosocial factors among the full sample and separately by age were examined in Table 2. Overall, perceived neighborhood disorder was significantly higher among individuals living in “mostly Black” neighborhoods compared to those living in “half Black” and “mostly White” communities (*p* < .05). In contrast, exposure to other social stressors was significantly lower among individuals from “mostly Black” and “half Black” neighborhoods relative to those living in “mostly White” areas (*p* < .05). While there were no significant differences in social support across PNRC, mastery, racial centrality, and COBP were highest among those in “mostly Black” communities (*p* < .05).

Among young adults, perceived neighborhood disorder was greatest for those in “mostly Black” neighborhoods (*p* < .05), and exposure to other social stressors was elevated among individuals in “half Black” communities (*p* < .05). At the same, no significant differences in mastery or social support emerged among young adults across PNRC, while racial centrality and COBP were significantly higher among those in “mostly Black” and “half Black” areas (*p* < .05).

For middle-aged adults, while perceived neighborhood disorder was higher for individuals in “mostly Black” neighborhoods (*p* < .05), there were no significant differences in exposure to other stressors. Mastery levels were similarly elevated among those in “mostly Black” and “mostly White” communities (*p* < .05), but there were no differences in social support or racial centrality levels. Individuals from “mostly Black” and “half Black” areas reported significantly higher levels of COBP compared to those from “mostly White” communities (*p* < .05).

Perceived neighborhood disorder was also highest among older adults who lived in “mostly Black” neighborhoods (*p* < .05), but these individuals also reported significantly lower exposure to other social stressors compared to older adults in “mostly White” communities (*p* < .05). There were no significant differences in mastery or social support across levels of PNRC. However, older adults who lived in mostly Black communities did report significantly higher levels of racial centrality (*p* < .05) and COBP (*p* < .05).

#### Psychosocial Risks and Resources, and Depressive Symptoms

##### Full Sample

Results from regression analyses for the full sample are shown in Table 3. Model 1 examined the direct association between PNRC and depressive symptoms. Relative to those living in predominately Black neighborhoods, individuals residing in communities characterized as “half Black” (b = 3.72, SE = 1.25, *p* < .01) or “mostly White” (b = 3.09, SE = 1.14, *p* < .001) experienced significantly more depressive symptoms; symptom levels for individuals living in these communities were statistically similar. Model 2 indicates that psychosocial risk factors were also significantly related to depressive symptoms, such that greater exposure to perceived neighborhood disorder (b = 0.11, SE = 0.05, *p* < .05) and other social stressors (b = 6.80, SE = 0.43, *p* < .001) were linked to significantly higher depressive symptoms. After accounting for risks, living in half Black and mostly White communities were still associated with significantly higher depressive symptoms levels, although these associations were reduced by more than 28% and 60%, respectively. Model 3 shows that higher levels of mastery (b = −0.71, SE = 0.09, *p* < .001), social support (b = −2.39, SE = 0.69, *p* < .001), and racial centrality (b = −0.42, SE = 0.21, *p* < .05) were associated with less frequent depressive symptoms; COBP was not directly related to depressive symptoms. After accounting for differences in these resources, the impact of living in half Black and mostly White communities on depressive symptoms was reduced by more than 32% and 41%, respectively. Psychosocial risks and resources were considered simultaneously in Model 4. While there were no longer significant differences in the depressive symptoms of individuals living in predominately Black versus predominately White communities, the influence of living in a “half Black” community on depressive symptoms was reduced by 58%. The full model explained 51% of the variance in depressive symptoms among Black Americans.

#### PNRC and Depressive Symptoms across Adulthood

##### Young Adults

The results for young adults are depicted in Table 4. Model 1 shows that living in “half Black” (b = 7.76, SE = 1.71, *p* < .001) and “mostly White” (b = 3.27, SE = 1.69, *p* < .05) communities were associated with significantly higher depressive symptom levels compared to living in “mostly Black” communities. Psychosocial risks and resources were considered in Model 2, and results indicate that while perceived neighborhood disorder not associated with depressive symptoms for young adults, exposure to other social stressors were significantly linked to greater symptom levels (b = 4.67, SE = 0.79, *p* < .001). In contrast, higher levels of mastery (b = −0.47, SE = 0.14, *p* < .01), social support (b = −2.98, SE = 0.95, *p* < .01), and racial centrality (b = −0.70, SE = 0.24, *p* < .01) were associated with fewer depressive symptoms. Nevertheless, more COBP contributed to higher symptom levels among young adults (b = 0.23, SE = 0.11, *p* < .05). Accounting for psychosocial risks and resources reduced the association between PNRC and depressive symptoms to non-significance, and these factors collectively explained 57% of the variation in depressive symptoms among Black young adults.

##### Middle-Aged Adults

PNRC was not significantly associated with depressive symptoms among middle-aged adults (Table 3, Model 3). Though perceived neighborhood disorder was also not linked to symptom levels, exposure to other social stressors was related to more depressive symptoms among this group (b = 6.60, SE = 1.03, *p* < .001). Of the four psychosocial resources examined, only mastery was significantly linked to depressive symptoms for middle-aged adults, such that greater mastery was associated with lower depressive symptom scores (b = −0.83, SE = 0.14, *p* < .001). Nonetheless, PNRC, psychosocial risks, and psychosocial resources collectively explain 55% of the variation in depressive symptoms among middle-aged Black adults.

##### Older Adults

PNRC was significantly associated with depressive symptoms among older adults, such that those living in predominately White communities had greater symptom levels than individuals living in predominantly Black communities (b = 4.80, SE = 1.42, *p* < .001; Model 6). However, residing in a racially integrated community was not linked to elevated symptoms. In Model 7, both perceived neighborhood disorder (b = 0.22, SE = 0.09, *p* < .05) and other social stressors (b = 4.74, SE = 0.67, *p* < .001) were related to significantly more symptoms. While mastery (b = −0.50, SE = 0.16, *p* < .01) and social support (b = −2.09, SE = 0.81, *p* < .05) contributed to fewer symptoms, racial centrality and COBP were not directly linked to depressive symptoms among older Black adults. PNRC, psychosocial risks, and psychosocial resources collectively explained 49% of the variation in depressive symptoms among this group.

## Discussion

The neighborhoods in which we live have major implications for health and psychological well-being. Beyond their physical conditions, community contexts pattern individuals’ exposure to health risks and their access to health-protective resources. Nevertheless, the specific processes through which communities influence mental health via psychosocial risks and resources among Black Americans has been less clear. To this end, the present study sought to clarify the role of PNRC, a key dimension of neighborhood context that reflects broader social processes such as residential segregation and the racialization of space (Anderson, 2015), in shaping mental health among Black Americans across adulthood. There were several notable findings.

To clarify the ways that community contexts pattern exposure to health risks and facilitate resources, we first assessed differences in psychosocial risks and resources across varied neighborhood compositions (i.e., neighborhoods perceived as mostly Black, half Black, or mostly White). Results show that PNRC contributes to both risks and resources for Black Americans. For example, individuals living in mostly Black communities reported elevated exposure to perceived neighborhood disorder, but higher access to mastery, racial centrality, and COBP relative to those living in mostly White neighborhoods. Living in a mostly Black area was also linked to lower levels of other social stressors, despite heightened perceived neighborhood disorder. Thus, it appears that the impact of PNRC is not “one size fits all,” rather it depends on the risk and resource factors considered. This distinction extends our understanding of PNRC among Black Americans. Specifically, our finding that individuals across all age groups who live in mostly Black communities report higher psychosocial resources challenges previous notions that predominately Black communities are inherently risky (Bécares et al., 2014; Do et al., 2019; Riley, 2018). In fact, the elevated levels of perceived neighborhood disorder observed point to the prevalence of structural and environmental challenges, rather than interpersonal stressors, among individuals living in these communities.

Findings from the present study also indicate that PNRC contributes to psychosocial risks and resources in distinct ways across age groups. For instance, in terms of risks, though perceived neighborhood disorder was consistently higher in mostly Black neighborhoods, exposure to other social stressors varied by age. Young adults living in half Black communities faced greater exposure to other stressors such as chronic stress, discrimination, and trauma. Exposure was much higher in White neighborhoods for older adults. There was no difference in exposure across contexts for middle-aged adults. Regarding resources, young and older adults living in mostly Black communities tended to endorse higher levels of racial centrality and COBP, whereas there were fewer differences in racial identity across contexts for middle-aged adults. By contrast, there were only variation in mastery across contexts for middle-aged adults, but not young or older adults. Taken together, these findings suggest that the impact of PNRC not only varies by risk or resource, but also depends on individuals’ life stage. This may be due to differences in other factors such as socioeconomic resources or life experiences. Prior research notes that individuals often develop more effective coping strategies to deal with challenges as they age (Umberson et al., 2014; Repetti, Taylor, & Seeman 2002). Relative to young people, older adults may also be more apt to draw on their historical knowledge and experiences and as a result, may be less influenced by their neighborhood context (Brondolo et al., 2009; Hughes et al., 2014). Nevertheless, since few studies have examined these relationships across age groups, additional research is needed to clarify the ways in which PNRC shapes psychosocial risks and resources across the life course. Second, we examined the relationship between PNRC and depressive symptoms. Overall, findings indicate that living in racially integrated (i.e., “half Black”) or predominately White neighborhoods was associated with greater depressive symptoms among Black Americans. This suggests that Black residents of predominately Black communities have better mental health than those living in more racially integrated or predominately White neighborhoods. This finding runs counter to the racial residential segregation hypothesis, which argues that limited socioeconomic resources within many predominately Black neighborhoods should heighten depressive symptoms among Black individuals (Bécares et al., 2014; Brenner et al., 2013; Diez Roux & Mair, 2010; Do et al., 2019). Instead, our findings are more in line with the ethnic density hypothesis, which asserts that Black Americans who live in predominately Black neighborhoods may, in fact, endorse fewer depressive symptoms because these contexts may provide greater availability of protective psychosocial resources (Bécares et al., 2014; Diez Roux & Mair, 2010). In other words, predominately Black neighborhoods may offer higher levels of social support, mastery, racial centrality for Black residents, indicating the importance of social relationships within these communities.

Third, we evaluated the extent to which psychosocial risks and resources account for the PNRC-depressive symptoms association among young, middle-aged, and older Black Americans. Results showed that not only did PNRC vary by age such that older adults were more likely to live in predominately White communities, but that the association between PNRC and depressive symptoms also differed across stages of adulthood. Among young adults, residing in racially integrated communities was associated with elevated symptoms, whereas living in predominately White neighborhoods was linked to worse symptoms for older adults. Young Black adults, who grew up in the 80s and 90s, experienced a public discourse of post-racial climate in the U.S., while also living through the Clinton Administration’s “War on Crime” policies (López, 2010). These historical contexts of criminalization of Black people in integrated and mostly White neighborhoods may shape how PNRC and depressive symptoms are negatively associated for the youngest population in this study (Jahn et al., 2021). PNRC was not associated with depressive symptoms for middle-aged Black Americans. Jim Crow laws, which began in the 1860s, legalized racial discrimination and segregation, and were overturned in 1964 by the Civil Rights Act. From a life course perspective, living through the Civil Rights Movement may have subsequently shaped older Black adults’ experiences, appraisals, and strategies for coping with stress, largely because they were coming of age and socialized in a time where outwardly hostile, overt racism was a common practice (Mouzon et al., 2017).

While psychosocial risks and resources explained a substantial portion of the PNRC-depressive symptoms association, analyses showed that the significance of these factors also varied significantly across age groups. For instance, social stressors and mastery influenced mental health for all groups, but perceived neighborhood disorder was only associated with poor mental health among older adults. This is particularly important, because physical characteristics of the neighborhood (e.g., vandalism, crime) may prevent older adults from accessing valuable social, physical, and institutional resources within the neighborhood, including senior centers, grocery stores, and parks (Glymour & Manly, 2008). Existing research has also shown that perceptions of physical disorder within community among older adults is associated with more chronic stress and negative health and behavioral outcomes, including lower likelihood of recovery from a mobility-related limitation, worse cognitive functioning, and reduced healthcare utilization (Latham-Mintus & Williams, 2015; Latham-Mintus, Vowels, & Chavan, 2020; Zaheed et al., 2019).

For young adults, all the resources we considered—mastery, social support, racial centrality, and COBP—were all influential resources. These findings are consistent with other studies that suggest that the health significance of psychosocial resources may vary throughout one’s life course, particularly as they move in and out of different neighborhood contexts (Hurd et al., 2013a; Pearlin et al., 2005). Given that these factors were not generally associated with mental health among middle-aged adults, additional research is needed to explore the distinct ways that this stage of adulthood may distinctly influence the mental health significance of PNRC, risks, and resources.

These results should be considered within the context of several limitations. The data for this study come from the Nashville Stress and Health Study, a regional sample from Nashville, Tennessee. While this urban city is recognized for having high racial tensions between police and racially minoritized communities, the data collection period (2011–2014) was further marked by several high-profile killings of unarmed Black Americans at the hands of law enforcement officials (Louie et al., 2021).Given the historical and contemporary experiences of racism and the distinct culture of race relations within the city of Nashville, additional research among a nationally representative sample within longitudinal data is needed to clarify the extent to which these patterns apply to other contexts and populations. Nevertheless, our results do provide important insights into the role of place-based psychosocial processes in shaping mental health among a socioeconomically diverse sample of Black American adults. Relatedly, the analyses presented here were cross-sectional. As such, we are unable to draw conclusions regarding temporal ordering and causality. Moreover, the measure of PNRC only provides one dimension of neighborhood context, based on respondent reports. The measure also only asks about the proportion of Blacks and Whites in the community, so the presence of other racial/ethnic groups was not considered. Future studies should also consider the ways that these relationships vary with SES, as recent studies suggest that SES may uniquely shape exposure to social stressors and the availability of psychosocial resources among Black Americans (Thomas Tobin & Moody, 2021). Finally, although we examine a wide array of psychosocial risks and resources in this study, we did not assess the potential interactions among these factors. Prior studies suggest that there may be synergistic relationships between neighborhood disorder and psychosocial resources such as mastery and social support (Gilster, 2016; Schieman, 2005). Thus, an important next step for future research would be to examine the ways that neighborhood contexts simultaneously produce psychosocial risks and resources and to evaluate the impact of these processes on psychological well-being.

Despite these limitations, findings from the present study collectively underscore the importance of neighborhood composition in understanding depressive symptoms among Black American adults across the life course. We demonstrate that living in racially integrated and predominately White neighborhoods generally is associated with elevated depressive symptoms among Black Americans, and while psychosocial risks and resources explained a substantial portion of this association, patterns varied significantly across age groups. Thus, this study highlights the importance of community contexts and individuals’ experiences within them, for shaping the mental health of this population. Moreover, our findings add key nuances to the stress process model by showing the pathways through which community contexts give rise to both psychosocial risks and resources that shape mental health among Black Americans. By clarifying these linkages, the present study enhances our understanding of the ways that PNRC shapes depressive symptoms among Black Americans across the life course and inform more effective strategies to reduce the burden of depression in this population. Thus, interventions aimed at minimizing psychosocial risks and enhancing resources within communities may be especially beneficial for improving Black American mental health outcomes across the life course.
